# SLC9A3 Protein Is Critical for Acrosomal Formation in Postmeiotic Male Germ Cells

**DOI:** 10.3390/ijms19010103

**Published:** 2017-12-29

**Authors:** Ya-Yun Wang, Han-Sun Chiang, Chiao-Yin Cheng, Yi-No Wu, Yung-Chih Lin, Hsuan-Che Liu, Wei-Kung Tsai, Yen-Lin Chen, Ying-Hung Lin

**Affiliations:** 1 Department of Chemistry, Fu Jen Catholic University, New Taipei City 242, Taiwan; vic0009@gmail.com (Y.-Y.W.); anthonypatho@gmail.com (Y.-L.C.); 2 Graduate Institute of Biomedical and Pharmaceutical Science, Fu Jen Catholic University, New Taipei City 242, Taiwan; 053824@mail.fju.edu.tw (H.-S.C.); chiaoyin810406@gmail.com (C.-Y.C.); timothy731027@msn.com (Y.-C.L.); brooke0972081233@gmail.com (H.-C.L.); 3 School of Medicine, Fu Jen Catholic University, New Taipei City 242, Taiwan; 133838@mail.fju.edu.tw; 4 Department of Urology, Mackay Memorial Hospital, Taipei 104, Taiwan; weiko11@gmail.com; 5 Department of Pathology, Cardinal Tien Hospital, New Taipei City 242, Taiwan

**Keywords:** SLC9A3, knockout mice, acrosome

## Abstract

Solute carrier family 9 isoform 3 (SLC9A3), a Na^+^/H^+^ exchanger, regulates the transepithelial absorption of Na^+^ and water and is primarily expressed on the apical membranes of the intestinal epithelium, renal proximal tubule, epididymis, and vas deferens. Loss of the *Slc9a3* allele in mice enhances intestinal fluid and causes diarrhoea as a consequence of diminished Na^+^ and HCO_3_^−^ absorption. Hence, the loss also causes male infertility and reveals the abnormal dilated lumen of the rete testis and calcification in efferent ductules. However, whether loss of *Slc9a3* alleles also disrupts mammalian spermatogenesis remains unknown. First, through immunoblotting, we determined that SLC9A3 is highly expressed in the murine testis compared with the small intestine, epididymis, and vas deferens. During murine spermatogenesis, SLC9A3 is specifically expressed in the acrosome region of round, elongating, and elongated spermatids through immunostaining. Furthermore, SLC9A3 signals are enriched in the acrosome of mature sperm isolated from the vas deferens. In *Slc9a3* knockout (KO) mice, compared with the same-aged controls, the number of spermatids on the testicular section of the mice progressively worsened in mice aged 20, 35, and 60 days. Sperm isolated from the epididymis of *Slc9a3* KO mice revealed severe acrosomal defects. Our data indicated that SLC9A3 has a vital role in acrosomal formation during spermiogenesis.

## 1. Introduction

### 1.1. Solute Carrier Family 9 Isoform 3 (SLC9A3)

SLC9A3 is one of nine plasma membrane Na^+^/H^+^ exchangers (SLC9A1-9) and is expressed on the apical membranes of the intestinal epithelium, renal proximal tubule, epididymis, and vas deferens [1,2,3,4,5,6,7]. The widely known functions of SLC9A3 are ion homeostasis regulation through Na^+^ and water absorption, and it often functionally couples with transepithelial Cl^−^/HCO_3_^−^ exchangers in the intestine [8]. Loss of the *Slc9a3* allele in mice results in increased intestinal fluid and diarrhoea because of decreased absorption of Na^+^ and HCO_3_^−^ [3]. The bioactivity of SLC9A3 at the apical sites of the epithelial membrane is regulated through the addition or removal of its phosphorylations, protein trafficking, and protein-protein interaction [7]. Recent studies have indicated that variations or mutations of *Slc9a3* are involved in the processes of several diseases (e.g., cystic fibrosis and congenital sodium diarrhoea) [9,10].

### 1.2. SLC9A3 and Male Reproductive Tract

First, the *Slc9a3* gene was identified in rats; it is mainly expressed in the intestine, stomach, and kidney [11]. In the male reproductive tract, SLC9A3 proteins are located at the apical sites of nonciliated cells in the effect ducts, which connect the rat testes and the principal cells of the epididymis to maintain the acidic luminal pH [5,12,13,14]. Zhou et al. determined that SLC9A3 is also expressed in the nonciliated cells of the efferent ducts in mice [15]. Furthermore, *Slc9a3^−^*^/*−*^ male mice become infertile with ageing and exhibit dilated rete testis and efferent ductules compared with controls [15]. Additionally, Zhou et al. determined that oestrogen action controls the expression levels of SLC9A3 and rate of Na^+^ transport in efferent ductules [15]. The main functions of SLC9A3 proteins in the male reproductive tract are fluid absorption and acidification [4,14,16].

### 1.3. Loss of SLC9A3 Allele Causes Obstructive Azoospermia-Like Phenotype

Mutated cystic fibrosis transmembrane conductance regulators (CFTRs) cause cystic fibrosis (CF), and most also result in congenital bilateral absence of the vas deferens (CBAVD) [17,18,19,20]. It is the major pathological cause of obstructive azoospermia [21]. However, CFTR mutations are absent in most Taiwanese patients with CBAVD; this is consistent with the low frequency of CF mutations in Asian populations [22]. Through oligonucleotide array-based comparative genomic hybridization (array-CGH), we identified the loss of one *Slc9a3* allele in 28.57% of Taiwanese men with CBAVD [23]. However, loss of SLC9A3 causes obstructive azoospermia and testicular atrophy [6]. Colleagues’ and our own studies have indicated that *Slc9a3^−/−^* adult male mice are completely infertile compared with wild-type (WT) and heterozygous mice [6,15]. *Slc9a3^−/−^* male mice possess an abnormally dilated lumen in the rete testis and calcification in the efferent ductules. Additionally, we identified damaged postmeiotic male germ cells in adult mice (>2 months old) [6]. The proposed pathological cause is efferent ductule obstruction. However, whether loss of the *Slc9a3* allele in mice also disrupts the spermatogenic process remains unknown. We sought to determine whether SLC9A3 expression is involved in mammalian spermatogenesis. In this study, we investigated the possible localization and functional roles of SLC9A3 during mammalian spermatogenesis through a KO mouse model.

## 2. Results

### 2.1. SLC9A3 is Specifically Expressed in Postmeiotic Male Germ Cells

**Figure 1:**
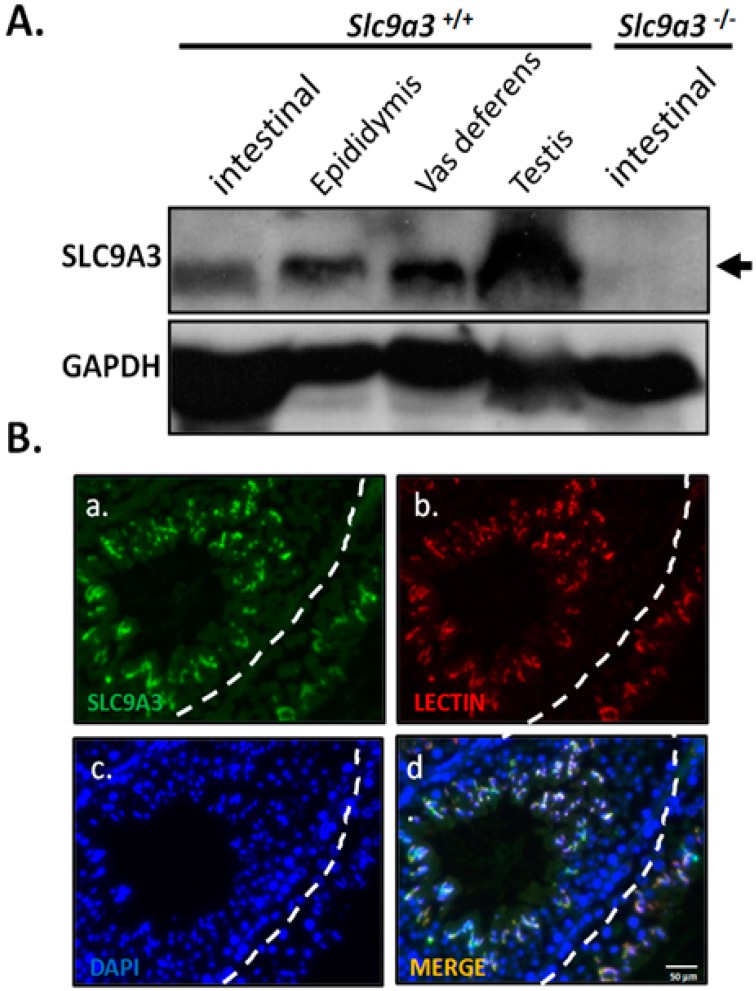
Solute carrier family 9 isoform 3 (SLC9A3) expression in the murine testis (**A**) SLC9A3 expression in the intestines, epididymides, vas deferentia, and testes of adult wild-type mice was compared with that in the intestines of *Slc9a3*^−/−^ mice through western blotting. The upper image shows SLC9A3 expression (**arrow**), and the lower image shows Glyceraldehyde 3-phosphate dehydrogenase (GAPDH) expression, which was used as a loading control; (**B**) Testis of WT mouse was detected through immunofluorescence staining with anti-SLC9A3 primary antibodies (SLC9A3 signals; **green**) (**a**). (**b**) Lectin (acrosome marker; **red**) and (**c**) 4′,6-diamidino-2-phenylindole (DAPI) (nucleus marker; **blue**) were costained and are displayed as a merged image (**d**). Scale bar = 50 μm.

The expressional patterns of SLC9A3 are restricted to several tissues (e.g., intestines, kidneys, epididymides, and vas deferentia) in rodents and humans [11]. To determine whether SLC9A3 expresses in testicular tissues, murine testicular tissues were evaluated through Western blotting. SLC9A3 is expressed in the murine intestine, epididymis, vas deferens, and testis in WT mice (Figure 1A, lanes 1–4; Supplementary Materials Figure S1). To evaluate the specificity of anti-SLC9A3 antibody, testicular samples from *Slc9a3*^−/−^ mice were used (Figure 1A, lane 5; Supplementary Materials Figure S2). Furthermore, testicular sections from adult mice were used to determine the localization. We determined that SLC9A3 is principally expressed in the postmeiotic male germ cells (Figure 1B and Supplementary Materials Figure S3). Through costaining with acrosomal marker (Lectin), we determined that SLC9A3 is specifically localized in the acrosomal regions of postmeiotic male germ cells.

### 2.2. SLC9A3 is Involved in Acrosomal Formations

**Figure 2:**
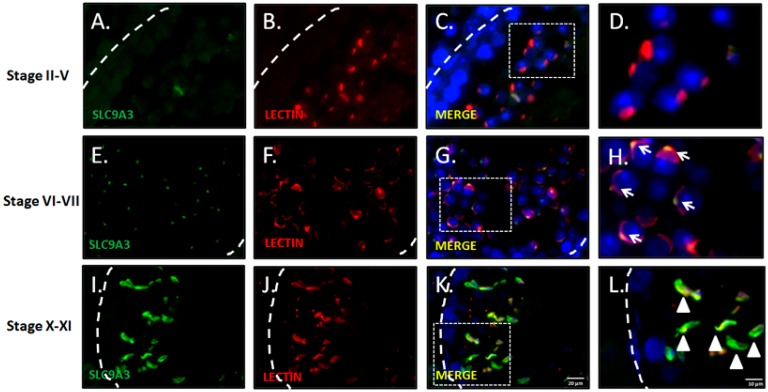
Expression patterns and localization of SLC9A3 during murine spermatogenesis. Top to bottom: Stages II–V (**A**–**D**); stages VI–VII (**E**–**H**); and stages X–XI (**I**–**L**) of spermatogenesis; (**A**,**E**,**I**) show the SLC9A3 signal (**green**); (**B**,**F**,**J**) show Lectin (**red**; acrosome marker); (**C**,**G**,**K**) merge from (**A**,**B**,**E**,**F**,**I**,**J**), respectively. The nucleus was stained using DAPI (**blue**). The enlarged Figure of (**C**,**G**,**K**) are shown in Figures (**D**,**H**,**L**), respectively. **Arrows** indicate the acrosomal vesicles. **Arrowheads** indicate the acrosome-covered regions of sperm heads. The scale bar is 20 μm in (**K**), and 10 μm in (**L**).

To determine the precise expressional stages of SLC9A3 during murine spermiogenesis, testicular sections and separated male germ cell populations were subject to immunofluorescence analysis. Figure 2 illustrates SLC9A3 expression in the acrosomal region at stages VI–VII of murine spermatogenesis (Figure 2E–H). Figure 2H depicts the specific granules vesicles of the acrosomal region. At stages X–XII, SLC9A3 covers the upper region of the sperm heads of the elongating spermatids (Figure 2I–L).

**Figure 3:**
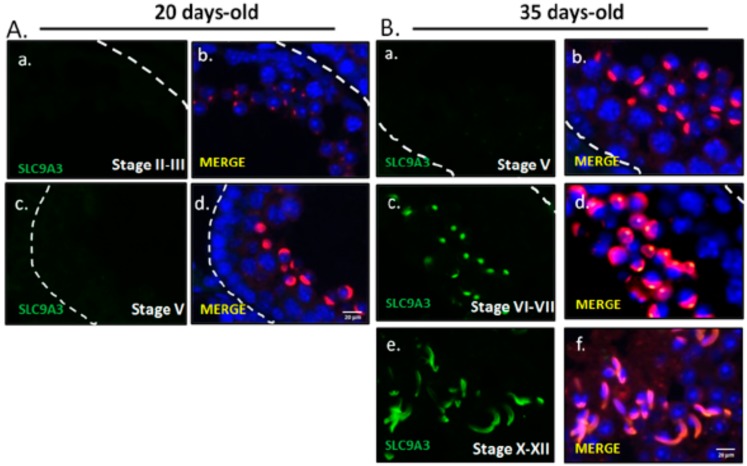
SLC9A3 starts to be expressed in 35 postnatal-day-old testes (**A**) Testicular sections prepared from 20-day-old mice. From top to bottom stages II–III (**a**,**b**) and stages V (**c**,**d**); (**B**) Testicular sections prepared from 35-day-old mice. From top to bottom: stages V (**a**,**b**), stages VI–VII (**c**,**d**), and stages X–XII (**e**,**f**). (**A**(**a**,**c**)) and (**B**(**a**,**c**,**e**)) indicated SLC9A3 signal (green). (**A**(**b**,**d**)) and (**B**(**b**,**d**,**f**)) indicated the merged figure with SLC9A3 signals (**green**), Lectin signals (**red**), and DAPI (**blue**). The scale bar is 20 μm in (**A**(**d**)) and (**B**(**f**)).

**Figure 4:**
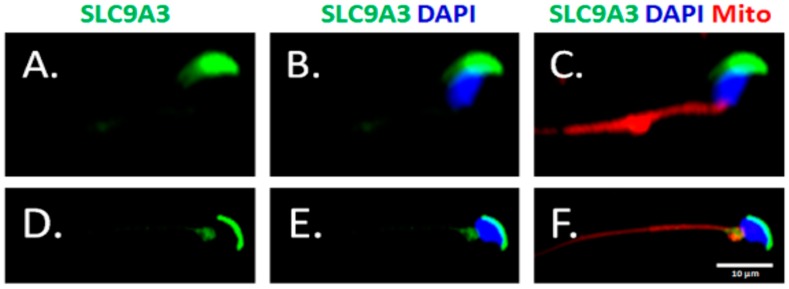
Expression patterns of SLC9A3 in elongating and elongated spermatids. Immunofluorescence detection of SLC9A3 in elongating spermatids (**A**–**C**) and elongated spermatids (**D**–**F**). (**A**,**D**) show SLC9A3 signals (**green**); (**B**,**E**) show SLC9A3 signals (**green**) and nucleus signals (**blue**); (**C**) is merged from (**A**,**B**), and MitoTracker signals (Mito; **red**); (**F**) is merged from (**D**,**E**), and MitoTracker signals (Mito; red). Scale bar = 10 μm.

To evaluate SLC9A3 expressed in the murine spermatocytes and the first waves of early-round spermatids, testicular sections from 20-day-old mice were used. The 20-day-old mice lacked SLC9A3 signals of testicular sections, which comprised spermatogonia, spermatocyte, and early-round spermatids, whereas 35-day-old mice exhibited strong expression of the testicular tissues, which had completed the first spermatogenic waves (Figure 3). During sperm-head formation, SLC9A3 covered the acrosomal regions of elongating (Figure 4A–C**)** and elongated spermatids (Figure 4D–F) isolated from the testicular tissue. These results indicated that SLC9A3 was specifically expressed for acrosomal formation in elongating and elongated spermatids.

### 2.3. Loss of Slc9a3 Allele Disturbs the Spermiogenic Process of 35-Day-Old Mice

**Figure 5:**
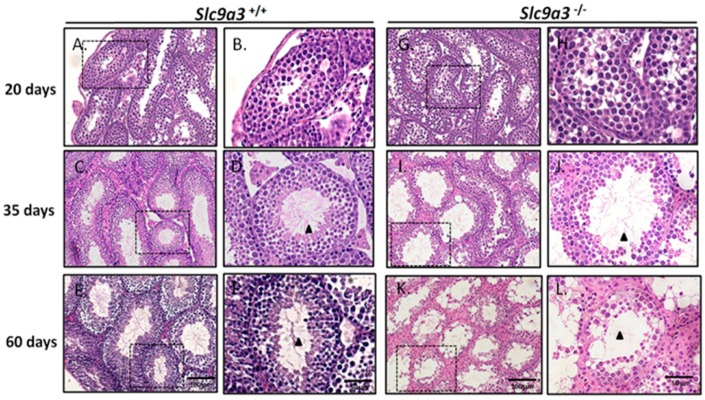
The testicular sections of 20-, 35-, and 60-day-old *Slc9a3* knockout mice. Comparison of the testicular sections of WT (**A**–**F**) and *Slc9a3*^−/*−*^ (**G**–**L**) mice according to H&E staining; (**B**,**D**,**F**,**H**,**J**,**L**) enlarged images from the areas boxed by a black dashed box in (**A**,**C**,**E**,**G**,**I**,**K**); Scale bar = 100 μm (**E**,**K**) and 50 μm (**F**,**L**); Elongated spermatids (**arrowhead**) observed in the ducts of the seminiferous tubules (**D**,**F**,**J**) but absent in (**L**).

To determine the early progressive effects of SLC9A3 deficiency, we evaluated the testicular sections of WT and *Slc9a3*^−/−^ mice at 20, 35, and 60 days old. The arrangements of the seminiferous tubules and interstitial tissue in the 20-, 35-, and 60-day-old WT mouse testes were effectively organized, and the male germ cells exhibited complete development (Figure 5A–F). The male germ cell population and numbers in seminiferous tubules of 20-day-old WT and *Slc9a3*^−/−^ mice were comparable (Figure 5G,H). First, in 35-day-old testes, spermatogenesis was moderately decreased and fewer spermatids were observed in the lumen compared with WT testes (Figure 5C,D,I,J; Arrowheads). Second, in 60-day-old mice, most testicular lumens in *Slc9a3*^−/−^ males lacked postmeiotic male germ cells and exhibited an inferior spermiogenic process (Figure 5K–L). The affected testicular sections of *Slc9a3*^−/−^ mice and the similar duration for SLC9A3 expression on acrosomal formation indicated that SLC9A3 was involved in acrosomal formation during the spermiogenic process.

### 2.4. SLC9A3 is Essential for Acrosome Integrity

**Figure 6:**
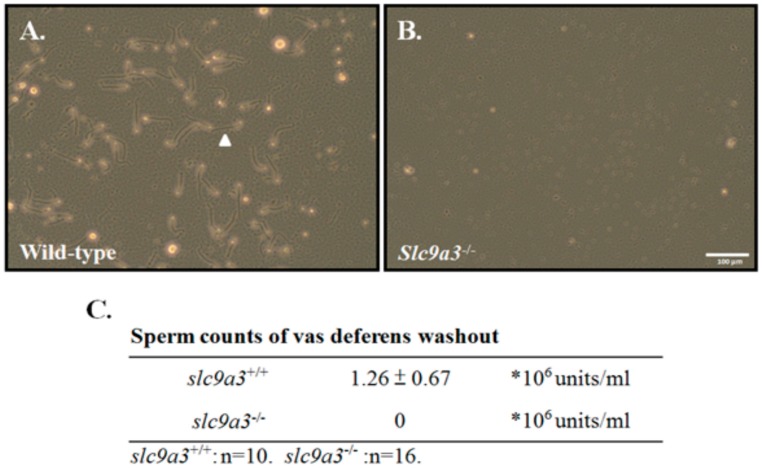
Sperm counts of vas deferens washout from *Slc9a3* knockout mice. Sperm were isolated from the vas deferens of (**A**) wild-type and (**B**) *Slc9a3* knockout mice. **Arrowhead** indicates mature sperm. Scale bar = 100 μm; (**C**) Sperm counts from *Slc9a3*^+/+^ (*n* = 10) and *Slc9a3*^−/−^ (*n* = 16) mice.

**Figure 7:**
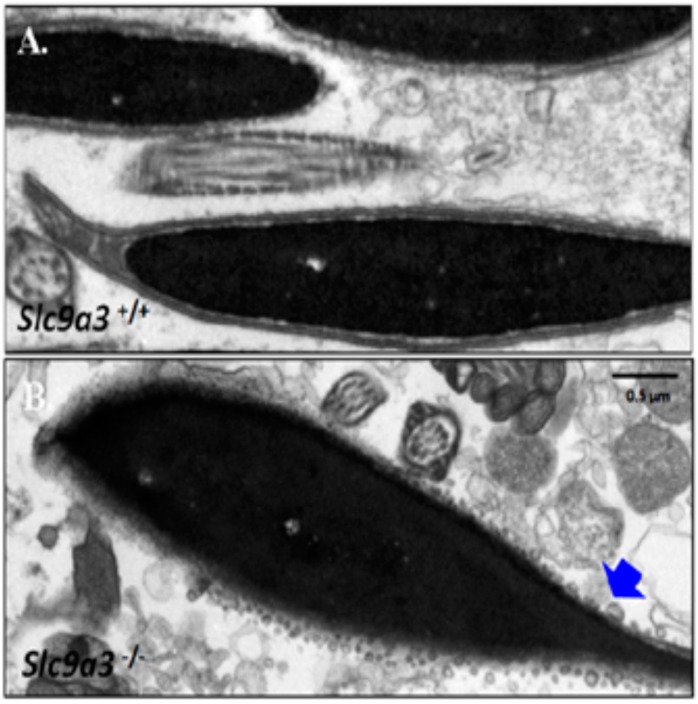
Ultrastructural defects of the sperm acrosome from *Slc9a3*^–/–^ mice. Sperm isolated epididymis of *Slc9a3*^+/+^ (**A**) and *Slc9a3*^−/−^ (**B**) mice. Electron micrograph showing the ultrastructure of sperm of 35-day-old WT (upper panel) and *Slc9a3*^−/−^ male mice (lower panel). **Arrow** indicates the acrosome defects in sperm from *Slc9a3*^−/−^ male mice. Scale bar = 0.5 μm.

To determine whether loss of SLC9A3 disrupts the terminal development and maturation of male germ cells, we evaluated the sperm counts and spermatozoa integrations from the vas deferens and epididymis. First, sperm was collected from the vas deferentia of WT (>60 days old, *n* = 10) and *Slc9a3*^−/−^ mice (>60 days old, *n* = 16). Figure 6 shows that the sperm of *Slc9a3*^−/−^ mice were absent, in contrast to WT mice. To determine the possible ultrastructure effects of SLC9A3 on sperm through transmission electron microscopy, tiny sperm were collected from the epididymis of the *Slc9a3*^−/−^ mice (*n* = 3) for comparison with the sperm of WT mice (*n* = 3). The acrosome from the sperm of the *Slc9a3*^−/−^ mice revealed a fragment-like structure, are similar to the small vesicles, in Figure 7B (indicated by the arrow), compared with the sperm of WT mice. These results indicate that the SLC9A3 function is critical to the integrity of sperm acrosome.

## 3. Experimental Section

### 3.1. Animals Preparation

The animal studies were approved by the Fu Jen Laboratory Animal Care and Use Committee (A10430). FVB.129(Cg)-*slc9a3^tm1Ges^*/J mice were obtained from Jackson Laboratory [3]. The genotyping of the *Slc9a3* allele was done through polymerase chain reaction (PCR) assay mixed with genomic DNA from the mouse tail. The genotyping primer was as follows: F1 (5′-CATACAACATAGGACTAGCC-3′), R1 (5′-CACTACTAGTCAGGCACTCT-3′) and R2 (5′-CACTACTAGTCAGGCACTCT-3′), as previous described [6]. More than 10 mice (*Slc9a3* KO and WT allele mice) were sacrificed by anaesthesia with isoflurane, and their intestines, epididymides, vas deferentia, and testes were collected.

### 3.2. Immunoblotting

The murine tissues were homogenised in a lysis buffer and total protein extractions were heated for 5 min at 37 °C before SDS-PAGE [24]. The antibodies against SLC9A3 (ab95299; Abcam, Cambridge, MA, USA) and GAPDH (G8795; Sigma-Aldrich, St. Louis, MO, USA) were applied and detected through chemiluminescence [6].

### 3.3. Histological Analysis and Immunofluorescence Analysis

At 20, 35, and 60 days of age, the WT (20-day-old: *n* = 5; 35-day-old: *n* = 3; 60 day-old; *n* = 3) and *Slc9a3*^−/−^ (20-day-old: *n* = 3; 35-day-old: *n* = 4; 60 day-old; *n* = 3) mice were sacrificed and their organs collected. The testes were fixed in Bouin’s solution (HT10132; Sigma-Aldrich, St. Louis, MO, USA) and processed to embed them in paraffin wax. Sections from these paraffin-embedded tissues were stained with haematoxylin and eosin (H&E) (Muto Pure Chemicals, Tokyo, Japan) for histological analysis. For immunofluorescence analysis, after dewaxing, sections were boiled with 0.1 M sodium citrate buffer (pH 6.0) for antigen retrieval. Sections were incubated overnight at 4 °C with diluted anti-SLC9A3 antibody (ab95299; Abcam, Cambridge, MA, USA). Furthermore, secondary antibodies, Alexa Fluor 488 (Invitrogen, Carlsbad, CA, USA), were used for against primary antibody and were costained with Lectin peanut agglutinin (acrosomal marker; l-32458; Invitrogen, Carlsbad, CA, USA) and 4′,6-diamidino-2-phenylindole (DAPI).

### 3.4. Separation of the Murine Male Germ Cell Populations

The male germ cells were isolated from the testicular tissues of adult mice (60-day-old; *n* = 3). As described in our previous article, male germ cells were separated depending on the density of the various male germ cell types through a centrifugal system [25]. Further, spermatogonia, spermatocyte, and spermatids were separated and dried on slides. Through MitoTracker staining, elongating and elongated spermatids (*n* > 10) were identified.

### 3.5. Electron Microscopy

Sperm were isolated from the caput epididymides from WT (60-day-old; *n* = 3) and *Slc9a3*^−/−^ mice (60-day-old; *n* = 3), and were directly fixed with 4% paraformaldehyde and 0.1% glutaraldehyde overnight at 4 °C. Subsequently, the sperm were washed with 0.1 M phosphate buffer (pH 7.2) and were rinsed with 1% osmium tetroxide at 25 °C for 2 h. After being re-treated with phosphate buffer, the samples were progressively dehydrated by raising the ethanol concentration. Subsequently, the sperm were embedded with Spurr’s resin kit (cat-14300; Electron Microscopy Sciences, Hatfield, PA, USA) overnight at 25 °C. The embedded samples were sectioned into 75-nm-thick sections using an ultramicrotome (EM UC7; Leica Microsystems, Wetzlar, Germany) and were mounted onto copper grids. Ultramicrographs were acquired using a transmission electron microscope (JEM-1400; JEOL, Tokyo, Japan) at 100 kVA.

## 4. Discussion

Studies have indicated that disrupted SLC9A3, a Na^+^/H^+^ exchanger, results in male sterility because of the disturbed acidification and obstructed male reproductive tract. In this study, we found that SLC9A3 is expressed in the acrosomal region of spermatids. Additionally, loss of SLC9A3 reduced sperm production in the testis and disrupted the acrosome formation in vivo. Our evidence suggests that SLC9A3 not only maintains the pH balance of the male reproductive tract but also facilitates acrosome development during murine spermiogenesis.

### 4.1. SLC9 Family Is Implicated in Sperm Formation

Maintaining and regulating intracellular pH (pHi) homeostasis are critical for cell physiology (e.g., regulating cell proliferation and cell survival) [26,27]. The SLC family is one of the major modelling protein families. The SLC9 family, including SLC9A, SLC9B, and SLC9C, are wildly expressed and extremely efficient in pHi regulation (Fuster et al. [28]). SLC9A1, SLC9A2, and SLC9A3 are enriched in the efferent ducts and regions of the epididymis [14]. Furthermore, SLC9A1, SLC9A5, and SLC9C1 (sNHE) are expressed in mature spermatozoa [16]. Three of these genes in the SLC9 family have been disrupted in mice. First, SLC9A1, located at the sperm midpiece and the disrupted *Slc9a1* allele in mice revels normal in male reproduction [29]. SLC9C1 is specifically expressed in the sperm principal piece, and loss of *Slc9c1* causes dramatic immotility of sperm and reduced male fertility [30]. Third, *Slc9a3* KO causes male infertility and reveals the abnormal dilated lumen of the rete testis and efferent ductules [15]. The major cellular function of SLC9A3 is transporting H^+^ in exchange for Na^+^ on the cell membrane, resulting in fluid absorption and acidification of the male reproductive tract [1]. In this study, we determined that the SLC9A3 expressed during acrosome formation and *Slc9a3* allele KO disrupted the acrosomal structure in mature sperm. We speculate that the loss of SLC9A3 protein disturbs fluid absorption and Na^+^ homeostasis during acrosome biogenesis, resulting in disintegration of the acrosomal membrane. This is first study to identify the SLC9 family’s involvement in acrosome formation and integration.

### 4.2. Oestrogen Receptor α Regulates SLC9A3 Expression in Male Reproduction

Zhou Qing et al. reported that oestrogen receptor α (ERα) may regulate the SLC9A3 mRNA expression in the efferent ductules by determining the SCL9A3 expression in an ERα knockout mice model [15]. Loss of ERα in mice resulted in testis weight reduction, lower sperm count, abnormal dilated lumen of the rete testis, and calcification in the efferent ductules [31,32]. These phenotypes are similar to those of *Slc9a3* knockout mice [6,15]. Recently, Joseph et al. revealed the high-frequency spontaneous acrosomal reactions of mature spermatozoa of ERα knockout mice [33]. In the present study, we characterized the SLC9A3 involved in sperm production and acrosomal integration of sperm. The similar results indicated that ERα may not only regulate SLC9A3 expression in the efferent ductules but also acrosomal development and maintenance.

### 4.3. SLC9A3-CFTR Complexes and Male Germ Cell Production

In a colleague’s study and our previous study, SLC9A3-CFTR formed a complex and disrupted the *Slc9a3* allele in mouse to decrease the CFTR levels in the epididymis and vas deferens [6,34]. In clinical studies, loss of CFTR resulted in CBAVD and obstructive azoospermia [17,18,20]. The CBAVD cases also reduced the success rates of fertilization in intracytoplasmic sperm injections using epididymal sperm [35,36]. Wu et al. found that CFTR is expressed in the human and mouse sperm head [37]. Furthermore, loss of CFTR reduces capacitation, disturbs the levels of pHi and cAMP production, and reduces membrane hyperpolarization of mouse sperm [37]. We speculate that loss of the *Slc9a3* allele in sperm production may also reduce CFTR, resulting in a disturbed intracellular pHi in sperm, and concluding in the disintegration of the membrane of the acrosome.

## 5. Conclusions

In this study, we determined that SLC9A3 also expresses in sperm and is critical for acrosomal integration. However, whether SLC9A3 affects the physiological functions of sperm (e.g., acrosome reaction and fertilizing capacity) remains to be investigated.

## Supplementary Materials

Supplementary materials can be found at www.mdpi.com/1422-0067/19/1/103/s1.
