# Supplementary material for: SLC9A3 Protein Is Critical for Acrosomal Formation in Postmeiotic Male Germ Cells
Source: Int J Mol Sci. 2017 Dec 29;19(1):103. doi: 10.3390/ijms19010103 (PMC5796053; doi:10.3390/ijms19010103)
Supplement: Supplementary file 1 [file ijms-19-00103-s001.pdf]

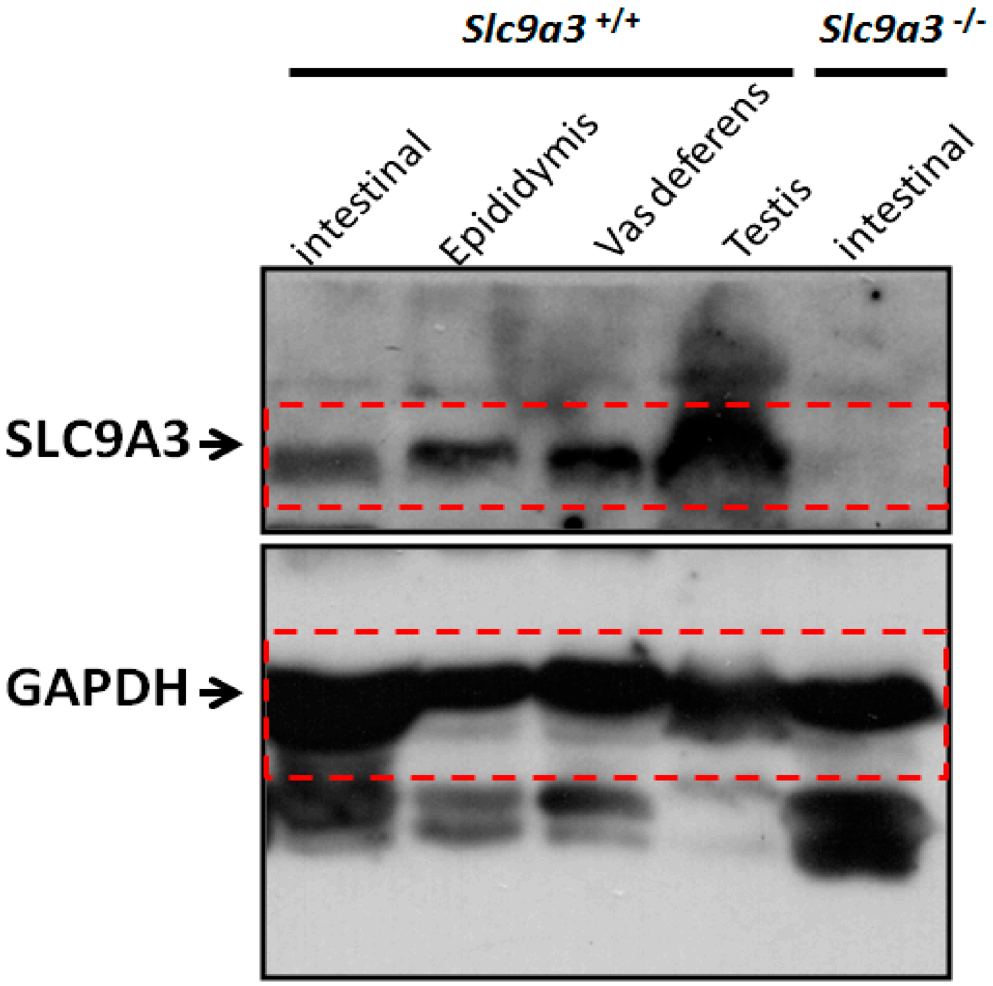

Supplemental Figure 1. SLC9A3 expression in the murine testis. SLC9A3 expressed in the intestines, epididymides, vas deferentia, and testes of adult wild-type mice compared with intestines of *Slc9a3*$^{-/-}$ mice was assessed through western blotting. The upper image shows SLC9A3 expression (arrow), and the lower image shows GAPDH expression, which was used as a loading control. The marked region is shown in Figure 1A.

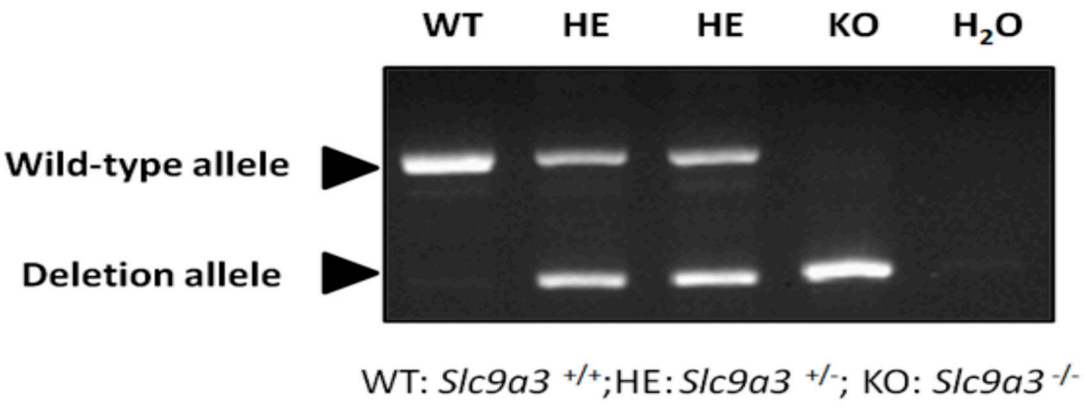

Supplemental Figure 2. Genotyping of SLC9A3 knockout mouse. Genotyping of *Slc9a3*$^{+/+}$ and *Slc9a3*$^{-/-}$ mice through PCR. Wild-type (WT): SLC9A3 $^{+/+}$; Heterozygous (HE): *Slc9a3*$^{+/-}$; Knockout (KO): *Slc9a3*$^{-/-}$. Control of PCR reaction (H_2O).

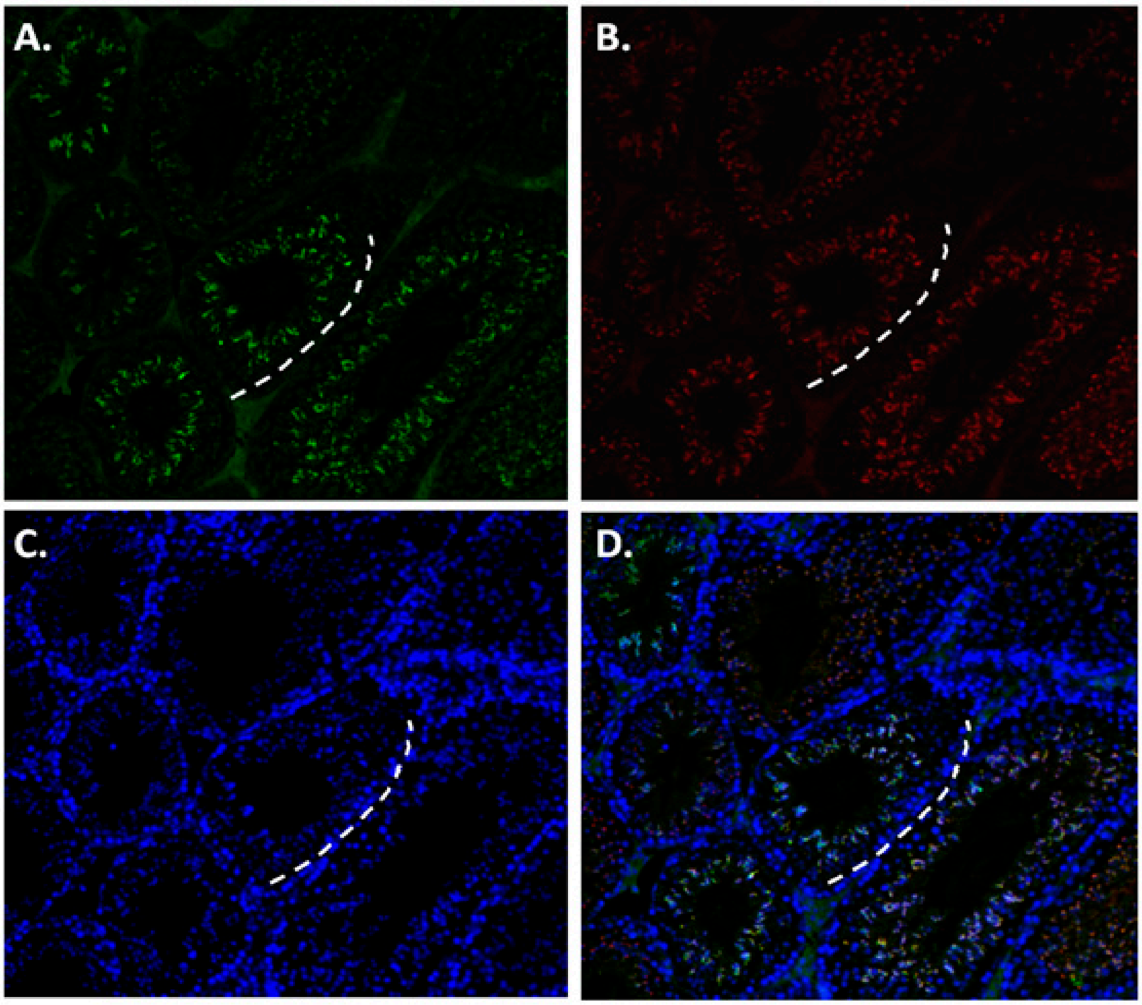

Supplemental Figure 3. SLC9A3 expression in the murine testis. The testis of WT mice was detected through immunofluorescence staining with anti-SLC9A3 primary antibodies (a.). (b.) Lectin (acrosome marker; red) and (c.) DAPI (nucleus marker; blue) are costained and displayed in merged images (d.). The marked region is shown in Figure 1B.
